# Therapeutic drug monitoring of infliximab compared to standard clinical treatment with infliximab: study protocol for a randomised, controlled, open, parallel-group, phase IV study (the NOR-DRUM study)

**DOI:** 10.1186/s13063-019-3734-4

**Published:** 2020-01-06

**Authors:** Silje W Syversen, Guro L Goll, Kristin K Jørgensen, Inge C Olsen, Øystein Sandanger, Johanna E Gehin, David J Warren, Joseph Sexton, Cato Mørk, Jørgen Jahnsen, Tore K Kvien, Nils Bolstad, Espen A Haavardsholm

**Affiliations:** 1 Department of Rheumatology, Diakonhjemmet Hospital, Box 23 Vinderen, 0319 Oslo, Norway; 2 Department of Gastroenterology, Akershus University Hospital, Sykehusveien 75, 1478 Lørenskog, Norway; 3 Research Support Services, Clinical Trial Unit, Oslo University Hospital, Postboks 4953 Nydalen, 0424 Oslo, Norway; 4 Section of Dermatology, Oslo University Hospital, Rikshospitalet, Postboks 4953 Nydalen, 0424 Oslo, Norway; 5 Department of Medical Biochemistry, Oslo University Hospital, Radiumhospitalet, Box 4953 Nydalen, 0424 Oslo, Norway; 6 Akershus Dermatology Center, Skårersletta 18, 1473 Lørenskog, Norway; 7 Faculty of Medicine, University of Oslo, Box 1089 Blindern, 0317 Oslo, Norway

**Keywords:** Therapeutic drug monitoring, Infliximab, Immunogenicity, Serum drug levels, Personalised medicine

## Abstract

**Background:**

Infliximab (INX) and other tumour necrosis factor inhibitors (TNFi) have revolutionised the treatment of several immune mediated inflammatory diseases. Still, many patients do not respond sufficiently to therapy or lose efficacy over time. The large interindividual variation in serum drug concentrations on standard doses and the development of anti-drug antibodies are thought to be major reasons for treatment failures. Therapeutic drug monitoring (TDM), an individualised treatment strategy based on systematic assessments of serum drug concentrations, has been proposed as a clinical tool to optimise efficacy of INX treatment. TDM seems reasonable both from a clinical and an economical point of view, but the effectiveness of this treatment strategy has not yet been demonstrated in randomised clinical trials. The NORwegian DRUg Monitoring study (NOR-DRUM) aims to assess the effectiveness of TDM, both with regard to the achievement of remission in patients starting INX treatment (part A) as well as to maintain disease control in patients on INX treatment (part B).

**Methods:**

The NOR-DRUM study is a randomised, open, controlled, parallel-group, comparative, multi-centre, national, superiority, phase IV study with two separate parts, NOR-DRUM A and NOR-DRUM B. Patients with rheumatoid arthritis, psoriatic arthritis, spondyloarthritis, ulcerative colitis, Crohn’s disease and psoriasis are included. In both study parts participants are randomised 1:1 to either TDM of infliximab (intervention group) or to standard treatment with infliximab without knowledge of drug levels or ADAb status (control group). NOR-DRUM A will include 400 patients starting INX therapy. The primary outcome is remission at 30 weeks. In NOR-DRUM B, 450 patients on maintenance treatment with INX will be included. The primary endpoint is occurrence of disease worsening during the 52-week study period.

**Discussion:**

As the first trial to assess the effectiveness, safety and cost-effectiveness of TDM in patients receiving TNFi for a range of immune mediated inflammatory diseases, we hope that the NOR-DRUM study will contribute to the advancement of evidence based personalised treatment with biological medicines.

**Trial registration:**

Clinicaltrials.gov, NCT03074656. Registered on 090317.

## Background

Infliximab (INX) and other tumor necrosis factor inhibitors (TNFi) have revolutionised the treatment of a range of prevalent chronic immune mediated inflammatory diseases and remission has become an achievable treatment goal. Unfortunately, more than half of the patients either do not respond sufficiently to therapy or lose efficacy over time [1–8]. A failure to achieve or maintain disease control has a major impact on patients’ quality of life and puts the individual at risk of developing irreversible organ damage and disability. Loss of clinical efficacy may be due to subtherapeutic drug levels [9–11]. Methods for measurement of serum drug concentrations of INX and other biological drugs have recently become available for use in clinical practice. In patients on standard doses of INX, significant interindividual variations in serum drug levels, ranging from undetectable to significantly above the presumed therapeutic range, have been revealed [9–11]. One major reason for this interindividual variation is the development of anti-drug antibodies (ADAb) which occur in 10%–60% of patients on INX [9–11]. The INX biosimilar CT-P13 has a similar immunogenicity profile to the innovator INX, and ADAb to INX are cross-reactive with CT-P13 [12–14]. Low levels of ADAb may be transient, but high levels influence the pharmacokinetics of the drug and decrease serum concentrations [9–11]. The presence of ADAb may also be associated with serious side effects of INX such as hypersensitivity reactions [9–11].

In an effort to optimise efficacy, clinicians often intensify the INX treatment by increasing the dose or decreasing the interval between infusions [8, 15, 16]. Large cohort studies show that up to 50% of patients have had one or more dose escalations within the first year of treatment [8, 15, 16]. The utility of empiric dose escalation is probably minimal in patients with high drug levels or ADAb, explaining the conflicting results regarding its effectiveness [15, 16]. Furthermore, the economic consequences of dosage increase is considerable given the high cost of INX.

With this background, therapeutic drug monitoring (TDM), an individualised treatment strategy based on systematic assessments of serum drug concentrations, has been proposed as a clinical tool to optimise efficacy, patient safety and cost-effectiveness of TNFi [17]. A treatment strategy based on TDM may improve INX therapy by minimising undertreatment, by reducing overtreatment, by allowing early identification of ADAb development, by enabling the detection of treatment failures, by timely discontinuation of ineffective treatment and by prevention of hypersensitivity reactions. In addition, TDM may also aid in choosing the next drug in patients where therapy has failed.

Although the exact therapeutic window of INX has yet to be clearly defined, a trough level > 3 μg/mL during maintenance therapy and > 20 μg/mL during the induction period has been associated with improved clinical outcomes in several observational studies and post hoc analyses of clinical trials across different diseases [18–26]. TDM has gained great interest within gastroenterology over recent years [27–29] and has now also been put on the research agenda in international rheumatology [30]. The European League Against Rheumatism (EULAR) has recently established a Task Force named “Therapeutic Drug Monitoring of Biopharmaceuticals in Rheumatology,” aiming to further develop the research agenda within this field. Supported by observational data [18–26] and clinical experience, TDM of INX treatment has already been implemented in clinical practice in some Norwegian and other European centres with available methodology and special interest in immunogenicity. Only two studies, however, have assessed the clinical effectiveness of TDM in a randomised controlled setting, both in inflammatory bowel disease (IBD) [24, 31]. In the TAXIT trial (Trough Level Adapted Infliximab Treatment study), dose optimisation increased the percentage of patients with Crohn’s disease (CD) in remission, but failed to show effectiveness of TDM during maintenance treatment [24]. TDM of INX treatment was also evaluated in the TAILORIX (A Randomised Controlled Trial Investigating Tailored Treatment With Infliximab for Active Luminal Crohn’s Disease) randomised controlled trial (RCT) [31]. In this trial, patients were randomised to either an algorithm based on clinical symptoms, biomarkers and TDM or to symptom-based management. No differences in the proportion of patients in steroid-free clinical and endoscopic remission (primary endpoint) were shown.

Data from RCTs to support guidelines and recommendations for implementation of TDM in standard care of patients on treatment with INX and other biological drugs are highly needed in an era of increasing use of therapeutic monoclonal antibodies. The main aim of the NORwegian DRUg Monitoring study (NOR-DRUM) is to assess the effectiveness of TDM, both in achieving remission in patients starting INX treatment (part A) as well as in maintaining disease control in patients on INX treatment (part B).

## Methods

### Overview of study design

**Fig. 1:**
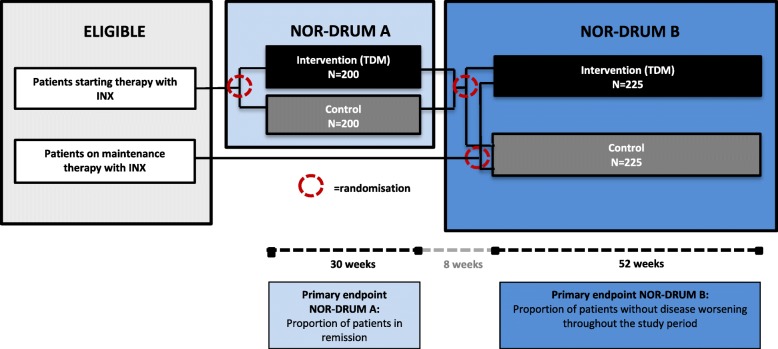
Overview of study design NOR-DRUM A and NOR-DRUM B. INX infliximab, NOR-DRUM Norwegian Drug Monitoring, TDM therapeutic drug monitoring

**Fig. 2:**
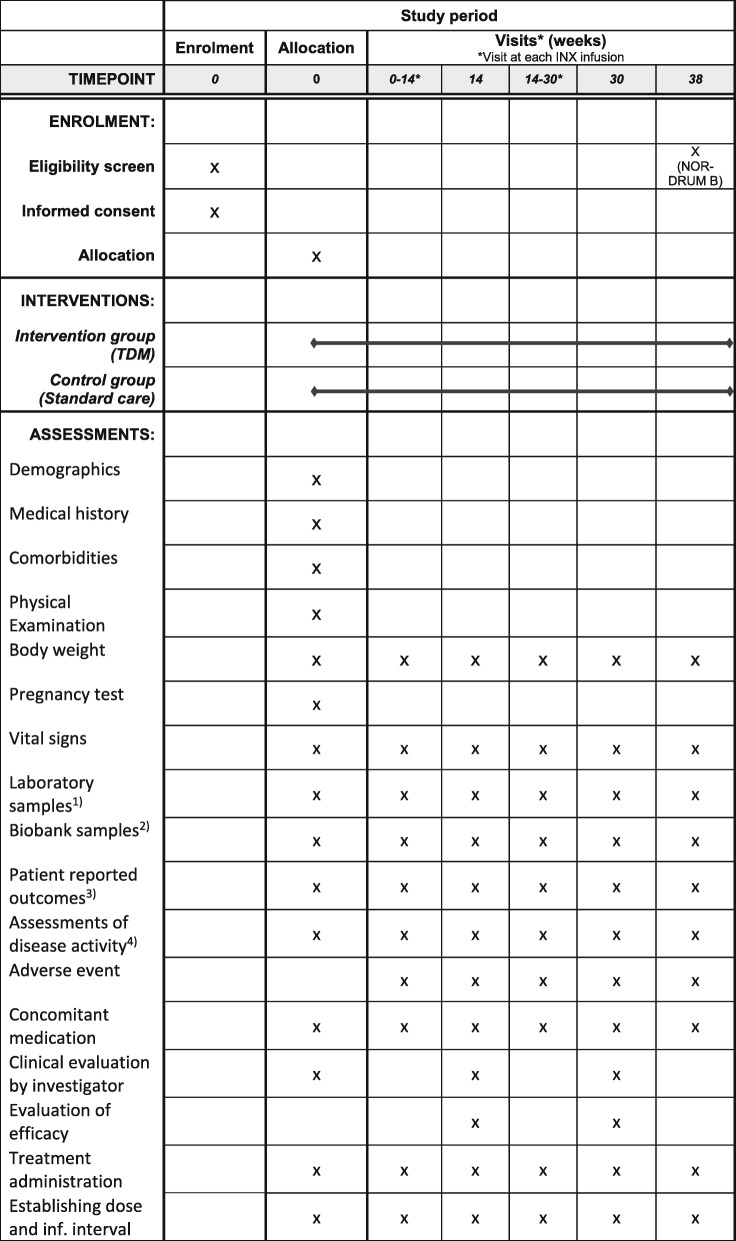
Schedule of enrolment, interventions and assessments NOR-DRUM A.1. Laboratory samples: haemoglobin, white blood cells with differentials, platelet counts, ALT, albumin, creatinine, CRP, ESR, fecal calprotectin (IBD only).2. Biobank samples: serum and full blood at baseline, serum at following visits.3. Patient-reported outcomes: patient global assessment of disease activity (NRS), EQ-5D, SF-36, WPAI-GH, RA: M-HAQ, RAID, PsA: M-HAQ, PsAID, DLQI, SpA: M-HAQ, BASDAI, UC and CD: IBDQ, Psoriasis: DLQI.4. Assessments of disease activity: Nurse/investigator global assessment of disease activity (VAS), RA: DAS28, CDAI, SDAI, PsA: DAS28, DAPSA, SpA: ASDAS, UC: Partial Mayo score, CD: HBI, Psoriasis: PASI. ALT alanine aminotransferase, ASDAS Ankylosing Spondylitis Disease Activity Score, BASDAI Bath Ankylosing Spondylitis Disease Activity Index, CD Crohn’s disease, CDAI Clinical Disease Activity Index, CRP C-reactive protein, DAS28 Disease Activity Score using 28 joints, DLQI Dermatology Life Quality Index, ESR erythrocyte sedimentation rate, HBI Harvey-Bradshaw Index, IBD inflammatory bowel diseases, IBDQ Inflammatory Bowel Disease Questionnaire, INX infliximab, MHAQ Modified Health Assessment Questionnaire, PASI Psoriasis Area and Severity Index, PMS partial Mayo score, Ps psoriasis, PsA psoriatic arthritis, PsAID Psoriatic Arthritis Impact of Disease, SDAI Simplified Disease Activity Index, RA rheumatoid arthritis, RAID Rheumatoid Arthritis Impact of Disease, SF-36 Short Form Health Survey, SpA spondyloarthritis, UC ulcerative colitis, VAS visual analogue scale

**Fig. 3:**
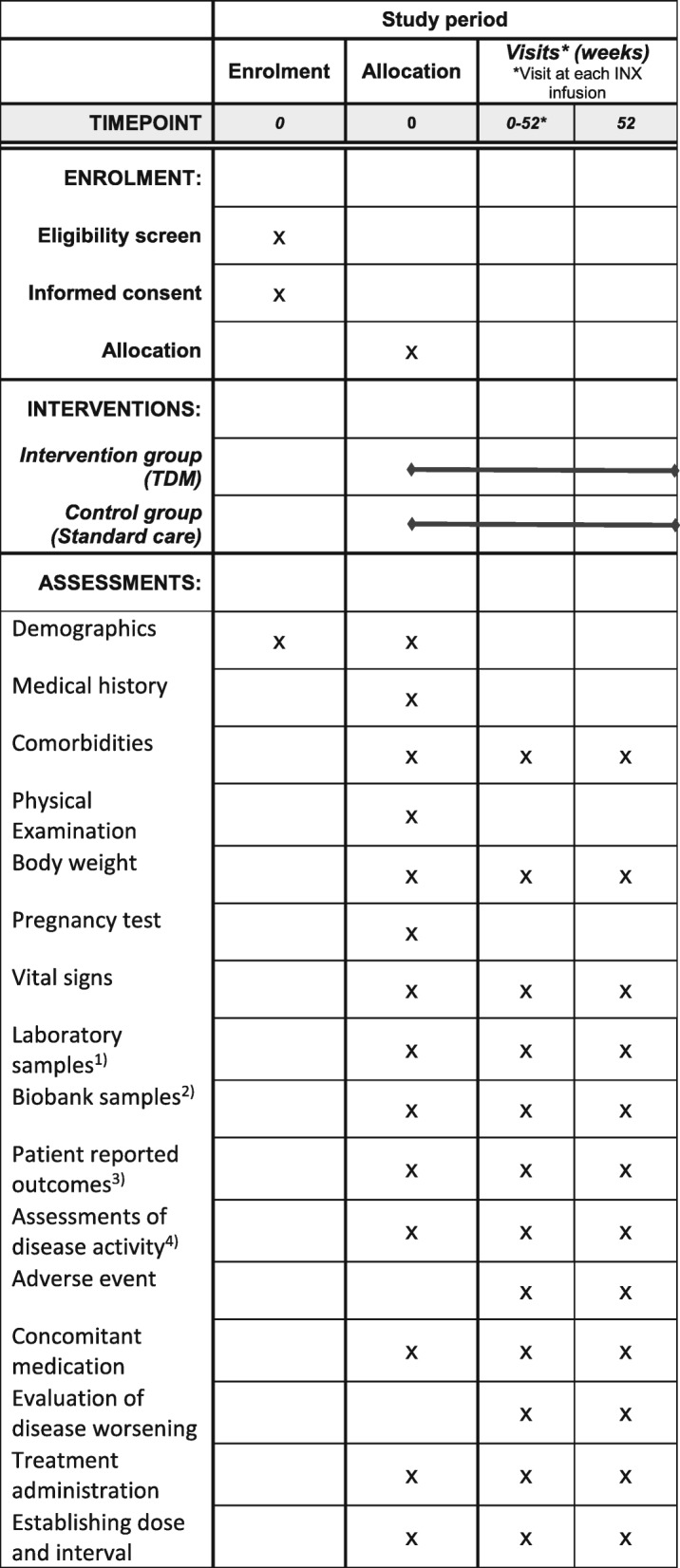
Schedule of enrolment, interventions and assessments NOR-DRUM B. 1. Laboratory samples: haemoglobin, white blood cells with differentials, platelet counts, ALT, albumin, creatinine, CRP, ESR, fecal calprotectin (IBD only).2. Biobank samples: serum and full blood at baseline, serum at following visits.3. Patient-reported outcomes: patient global assessment of disease activity (NRS), EQ-5D, SF-36, WPAI-GH, RA: M-HAQ, RAID, PsA: M-HAQ, PsAID, DLQI, SpA: M-HAQ, BASDAI, UC and CD: IBDQ, Psoriasis: DLQI.4. Assessments of disease activity: Nurse/investigator global assessment of disease activity (VAS), RA: DAS28, CDAI, SDAI, PsA: DAS28, DAPSA, SpA: ASDAS, UC: Partial Mayo score, CD: HBI, Psoriasis: PASI. ALT alanine aminotransferase, ASDAS Ankylosing Spondylitis Disease Activity Score, BASDAI Bath Ankylosing Spondylitis Disease Activity Index, CD Crohn’s disease, CDAI Clinical Disease Activity Index, CRP C-reactive protein, DAS28 Disease Activity Score using 28 joints, DLQI Dermatology Life Quality Index, ESR erythrocyte sedimentation rate, HBI Harvey-Bradshaw Index, IBD inflammatory bowel diseases, IBDQ Inflammatory Bowel Disease Questionnaire, INX infliximab, MHAQ Modified Health Assessment Questionnaire, PASI Psoriasis Area and Severity Index, PMS partial Mayo score, Ps psoriasis, PsA psoriatic arthritis, PsAID Psoriatic Arthritis Impact of Disease, SDAI Simplified Disease Activity Index, RA rheumatoid arthritis, RAID Rheumatoid Arthritis Impact of Disease, SF-36 Short Form Health Survey, SpA spondyloarthritis, UC ulcerative colitis, VAS visual analogue scale

The NOR-DRUM study is a randomised, controlled, parallel-group, open, comparative, multi-centre, national, superiority, phase IV study with two separate parts (NOR-DRUM A and NOR-DRUM B) comparing TDM of INX treatment to standard INX treatment. The study design is outlined in Fig. 1. The schedule of enrolment, interventions and assessments is given in Fig. 2 (NOR-DRUM A) and Fig. 3 (NOR-DRUM B). The Standard Protocol Items: Recommendation for Interventional rials (SPIRIT) checklist detailing the items in this clinical trial protocol is provided as Additional file 1.

### Study setting and population

All Norwegian hospitals treating patients with rheumatoid arthritis (RA), psoriatic arthritis (PsA), spondyloarthritis (SpA), ulcerative colitis (UC), Crohn’s disease (CD) or psoriasis (Ps) are invited to participate. The study is conducted at 21 study centres distributed across all four Norwegian health regions. After initiation of the sites, potential study participants (patients who are either starting INX or who have been treated with INX for a minimum of 30 weeks or maximum of three years) are informed about the study by their treating physician. To maintain a high rate of enrolment, the study lead and the clinical coordinators (one rheumatologist, one gastroenterologist and one dermatologist) are in frequent contact with the local principal investigators (PIs) and study nurses, hold national investigators meetings and frequently send out newsletters. After giving informed consent, patients are screened and, if eligible, included in the study by study personnel. Inclusion and exclusion criteria are shown in Table 1. Recruitment of patients is taking place in a competitive manner until 400 patients have been included in NOR-DRUM A and 450 patients have been included in NOR-DRUM B. As the study visits are carried out according to the patient’s INX treatment schedule, we expect a high rate of retention in the NOR-DRUM trial. If a patient is missing for scheduled INX infusion, the study nurse will contact the patient and ensure that a new appointment for infusion/study visit is scheduled.

**Table 1:** Eligibility criteria

Inclusion criteria	
NOR-DRUM A	
All of the following conditions must apply to the prospective patient at screening; 1. A clinical diagnosis of one of the following: RA, SpA, PsA^a^, UC, CD or Ps 2. Male or non-pregnant female 3. Age ≥ 18 and < 75 years at screening 4. A clinical indication to start INX 5. Patient not in remission according to diagnosis-specific disease activity scores 6. Patient capable of understanding and signing an informed consent form	
NOR-DRUM B	
All of the following conditions must apply to the prospective patient at screening; 1. A clinical diagnosis of one of the following: RA, SpA, PsA^a^, UC, CD or Ps 2. Male or non-pregnant female 3. Age ≥ 18 and < 75 years at screening 4. On maintenance therapy with INX for a minimum of 30 weeks and a maximum of three years 5. A clinical indication for further INX treatment 6. Patient capable of understanding and signing an informed consent form	
Exclusion criteria	
NOR-DRUM A	
A patient will be excluded from the study if they meet any of the following criteria: 1. Major co-morbidities, such as previous malignancies within the last five years, severe diabetes mellitus, severe infections, uncontrollable hypertension, severe cardiovascular disease, severe respiratory diseases, demyelinating disease, significant chronic widespread pain syndrome, laboratory abnormalities/significant renal or hepatic disease and/or other diseases or conditions where treatment with INX is either found contra-indicated by the clinician or which make adherence to the protocol difficult 2. A positive screening for tuberculosis or viral hepatitis 3. Inadequate birth control, pregnancy or patient considering becoming pregnant during the study period 4. Psychiatric or mental disorders, alcohol abuse or other substance abuse, language barriers or other factors which makes adherence to the study protocol difficult 5. Prior use of INX within the last six months 6. For patients with UC and CD: functional colostomy or ileostomy or extensive colonic resection with < 25 cm of the colon left in situ	
NOR-DRUM B	
A patient will be excluded from the study if they meet any of the following criteria: 1. Major co-morbidities, such as previous malignancies within the last five years, severe diabetes mellitus, severe infections, uncontrollable hypertension, severe cardiovascular disease, severe respiratory diseases, demyelinating disease, significant chronic widespread pain syndrome, laboratory abnormalities/significant renal or hepatic disease and/or other diseases or conditions where treatment with INX is either found contra-indicated by the clinician or which make adherence to the protocol difficult 2. Inadequate birth control, pregnancy or patient considering becoming pregnant during the study period 3. Psychiatric or mental disorders, alcohol abuse or other substance abuse, language barriers or other factors which makes adherence to the study protocol difficult 4. For patients with UC and CD: functional colostomy or ileostomy. Extensive colonic resection with < 25 cm of the colon left in situ.	

^a^PsA with predominantly axial manifestations should be included and assessed as SpA

*CD* Crohn’s disease, *INX* infliximab, *Ps* psoriasis, *PsA* psoriatic arthritis, *RA* rheumatoid arthritis, *SpA* spondyloarthritis, *UC* ulcerative colitis

### Randomisation procedures and allocation

Eligible patients are assigned a unique patient identification number. In NOR-DRUM A, patients are allocated in a 1:1 ratio between intervention and control, using a computer randomisation procedure stratifying by diagnosis (RA, SpA, PsA, UC, CD, Ps). The randomisation is blocked within each stratum. In NOR-DRUM B, patients are allocated in a 1:1 ratio between intervention and control, using a computer randomisation procedure stratifying by diagnosis (RA, SpA, PsA, UC, CD, Ps) as well as: (1) by study arm (intervention or control) if the patient originates from NOR-DRUM A; or (2) by prior or no prior TDM in the clinic (defined as one or more assessments of serum drug level during the last three infusions) if the patient originates from NOR-DRUM B. The randomisation is blocked within each stratum. The computer-generated randomised allocation sequence is imported into the electronic case report form (eCRF) system and made available to site personnel. The allocation is not available until the patient has signed the informed consent form, deemed eligible to participate and entered in the eCRF. Authorised personnel will only know the allocation of included patients, but not for future patients. Details of block size and allocation sequence generation are kept unavailable to those who enrol patients or assign treatment.

### Intervention

In both study parts (A and B), patients are randomised to either:

1. Administration of INX according to a treatment strategy based on TDM and assessments of ADAb (intervention group);
2. Administration of INX according to standard clinical care, without knowledge of drug levels or ADAb status (control group).

**Fig. 4:**
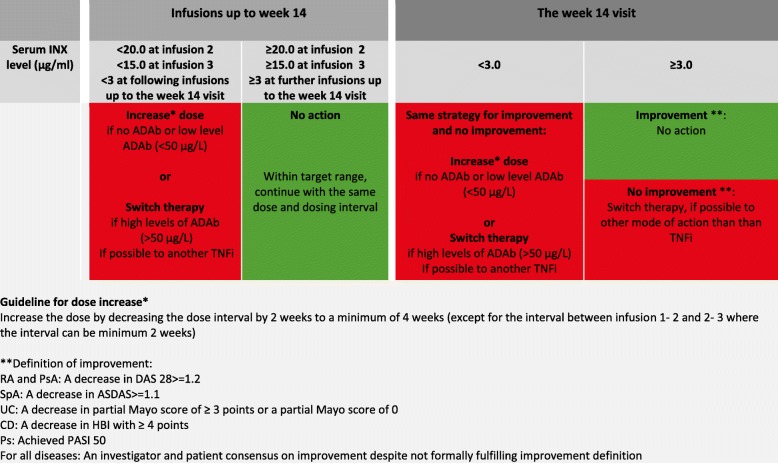
Algorithm for administration of INX in NOR-DRUM A (visits ≤ week 14), intervention group. ADAb anti-drug antibody(ies), ASDAS Ankylosing Spondylitis Disease Activity Score, BASDAI Bath Ankylosing Spondylitis Disease Activity Index, CD Crohn’s disease, DAS28 Disease Activity Score using 28 joints, HBI Harvey-Bradshaw Index, INX infliximab, PASI Psoriasis Area and Severity Index, PsA psoriatic arthritis, RA rheumatoid arthritis, SpA spondyloarthritis, UC ulcerative colitis

**Fig. 5:**
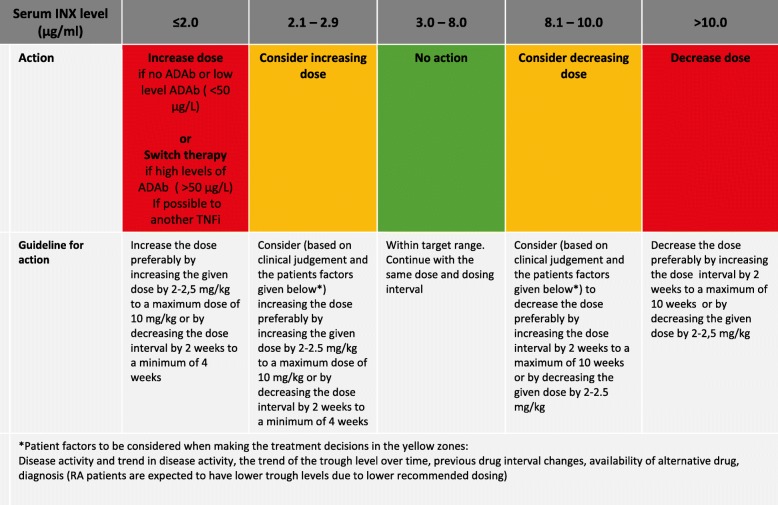
Algorithm for administration of INX NOR-DRUM A (visits ≥ 14 weeks) and NOR-DRUM B, intervention group. ADAb anti-drug antibody(ies), ASDAS Ankylosing Spondylitis Disease Activity Score, BASDAI Bath Ankylosing Spondylitis Disease Activity Index, CD Crohn’s disease, DAS28 Disease Activity Score using 28 joints, HBI Harvey-Bradshaw Index, INX infliximab, PASI Psoriasis Area and Severity Index, PsA psoriatic arthritis, RA rheumatoid arthritis, SpA spondyloarthritis, UC ulcerative colitis

The treatment strategy in the intervention group is outlined in Figs. 4 and 5. At each visit/infusion, serum levels of INX (s-INX) and ADAb are assessed; in the intervention group, the levels are reported back to the investigators who will adjust the dose or infusion interval according to the strategy (Figs. 4 and 5). During the first infusions (up to and including week 14), the dose is adjusted by decreasing the infusion interval (Fig. 4). After week 14, the INX dose or interval can be increased or decreased to reach the target range of 3–8 μg/mL (Fig. 5).

The randomised treatment strategy is continued for the whole study period (38 [±4] weeks in NOR-DRUM A and 52 [±4] weeks in NOR-DRUM B) with study visits at each scheduled INX infusion. After 38 weeks in NOR-DRUM A, patients who are still on INX are included and re-randomised in NOR-DRUM B. Patients who, for any reason (lack of efficacy, side effects or other) are switched to another treatment during the study, will still be followed with study visits according to the intentional infusion intervals and remain in the allocated group. Immunosuppressive concomitant treatments initiated before inclusion in the study are continued. To improve compliance to the strategy in the intervention group, an interactive eCRF with guidelines for INX dosing based on levels of INX have been developed (Viedoc 4™, Uppsala, Sweden).

In NOR-DRUM A, improvement is assessed after three months (week 14 visit), for which a separate algorithm is used (Fig. 4). Improvement is defined as: RA and PsA: a decrease in Disease Activity Score using 28 joints (DAS 28-SR) of ≥ 1.2 from baseline; SpA: a decrease in Ankylosing Spondylitis Disease Activity-C-reactive protein Score (ASDAS-CRP) of ≥ 1.1 from baseline; UC: a decrease in the partial Mayo score of ≥ 3 from baseline or a partial Mayo score of 0; CD: a decrease in the Harvey-Bradshaw (HBI) of ≥ 4 from baseline; Ps: Psoriasis Area and Severity Index (PASI) 50 (a 50% reduction in the PASI score from baseline); investigator and patient consensus on improvement: if a patient does not fulfil the formal definition, but both the patient and the investigator agree that the patient has improved this should be considered as improvement but recorded separately in the CRF.

Patients have the right to withdraw from the study at any time for any reason. In case a patient decides to do so, they will be asked if they can still be contacted for further information, so that a final evaluation can be made with an explanation for the withdrawal, including assessment of possible adverse events (AE).

The investigator may discontinue the patient from further study participation if such participation will put the patient at risk of medical injury or there has been a major protocol violation.

### Outcomes

#### Primary outcomes

The primary outcome in NOR-DRUM A is remission at week 30, while the primary outcome in NOR-DRUM B is sustained disease control without disease worsening throughout the study period. Remission and disease worsening are defined as disease-specific activity scores as summarised in Table 2.

**Table 2:** Definition of primary outcomes

Disease	Disease activity scoring tool	Value defining remission	Values defining disease worsening
RA	Disease Activity Score using 28 joint (DAS 28)	< 2.6	Increase ≥ 1.2 from inclusion and a minimum score of 3.2
PsA	Disease Activity Score using 28 joint (DAS 28)	< 2.6	Increase ≥ 1.2 from inclusion and a minimum score of 3.2
SpA	Ankylosing Spondylitis Disease Activity Score with CRP (ASDAS-CRP)	< 1.3	Increase of ≥ 1.1 from inclusion and a minimum of 2.1
CD	Harvey-Bradshaw Index (HBI) score	≤ 4	Increase of ≥ 3 points from inclusion and a minimum partial score of 5 points
UC	Partial Mayo score	≤ 2 with no sub scores > 1	Increase of ≥ 3 points from inclusion and a minimum partial score of 5 points
Ps	Psoriasis Area and Severity Index (PASI)	≤ 4	Increase ≥ 4 points from inclusion and a minimum score of 7 points

*CD* Crohn’s disease, *Ps* psoriasis, *PsA* psoriatic arthritis, *RA* rheumatoid arthritis, *SpA* spondyloarthritis, *UC* ulcerative colitis

Additionally, disease worsening can be recorded based on patient and investigator consensus: if a patient does not fulfil the formal definition, but experiences a clinically significant worsening according to both the investigator and patient that leads to a major change in treatment (i.e. switching from INX to another immunosuppressant/Disease-Modifying Anti-Rheumatic Drug [DMARD], adding a immunosuppressant/DMARD, increasing the dose of a concomitant immunosuppressant/DMARD, adding systemic glucocorticoids [po., iv. or im.], receiving more than one i.a. glucocorticoid injection at one visit or increasing INX for clinical reasons), this should be considered as a disease worsening but be recorded separately in the eCRF.

#### Secondary and exploratory outcomes

In NOR-DRUM A, secondary outcomes include generic outcomes: time to sustained remission (a status of remission on all consecutive visits following the initial obtained remission); patient’s and physician’s global assessment of disease activity; biochemical parameters of disease activity; occurrence of ADAb; occurrence of and reason for drug discontinuation; safety and cost-effectiveness; utility and quality of life in addition to disease specific activity composite scores assessed at all visits: RA, DAS28, Clinical Disease Activity Index (CDAI), Simplified Disease Activity Index (SDAI), Rheumatoid Arthritis Impact of Disease (RAID), Modified Health Assessment Questionnaire (MHAQ); PsA, DAS28, Psoriatic Arthritis Impact of Disease (PsAID) score-9, Disease Activity Psoriatic Arthritis (DAPSA) score, MHAQ, Dermatology Life Quality Index (DLQI); SpA, ASDAS, Bath Ankylosing Spondylitis Disease Activity Index (BASDAI), MHAQ; UC, Partial Mayo Score, Inflammatory Bowel Disease Questionnaire (IBDQ); CD, HBI, IBDQ; Ps, PASI, DLQI. In NOR-DRUM B secondary outcomes include generic outcomes: time to disease worsening; patient and physician global assessment of disease activity; biochemical parameters of disease activity; occurrence of ADAb; occurrence of and reason for drug discontinuation; safety and cost–effectiveness; utility and quality of life in addition to disease specific activity scores assessed at all visits as listed above for NOR-DRUM A.

### Study schedule and assessments

The schedule of enrolment, interventions and assessments in NOR-DRUM A and NOR-DRUM B is depicted in the SPIRIT Figs. 2 and 3. The study visits are carried out according to the patient’s INX treatment schedule and the number of visits varies depending on the infusion intervals. Extra study visits are arranged at the request of the patient and/or the investigator. If INX treatment is terminated, patients are assessed according to the original infusion plan (every eight weeks). The assessments performed at each visit are shown in Figs. 2 and 3. The primary outcome in NOR-DRUM A (remission) will be recorded at the week-30 (±2 weeks) visit. In NOR-DRUM B, the primary outcome (occurrence of disease worsening) is recorded at every visit during the 12 month follow-up period. If the patients perceive increased disease activity, a non-scheduled visit will be arranged within one week in order to identify occurrence of disease worsening.

### Laboratory assessments

Blood samples are collected at all visits before the infusion. Hematology and clinical chemistry parameters, as well as acute phase reactants and faecal calprotectin in patients with IBD, are analysed at the local hospital laboratory or referred to other laboratories according to local practice. Samples for biobanking and measurement of serum INX levels and ADAb are sent to the Department of Medical Biochemistry, Oslo University Hospital, Radiumhospitalet. Serum INX levels (trough) and ADAb are measured using in-house assays automated on the AutoDELFIA immunoassay platform (PerkinElmer, Waltham, MA, USA) [32]. Laboratory data are stored in the laboratory information system; results for patients in the intervention group are reported to the local investigator. All results will be transferred to the PI upon completion of the study.

### Statistics

#### Sample size and power considerations

Sample sizes are determined for each of the two study parts separately. NOR-DRUM A: under the assumption of an (absolute) increase in remission rate of 15% (from 40% to 55%), 358 completed patients are needed in order to reject the null hypothesis at a 5% significance level with 80% power. Adjusting for possible drop-outs, we plan to randomise 400 patients.

NOR-DRUM B: under the assumption of an (absolute) decrease in proportion of patients with disease worsening of 12.5% (from 30% to 17.5%), 414 completed patients are needed in order to reject the null hypothesis at a 5% significance level with 85% power. Adjusting for possible drop-outs, we plan to randomise 450 patients.

#### Statistical plan

Separate statistical analysis plans (SAP) for each study part will provide further details on the planned statistical analyses. The SAPs will be finalised, signed and dated before data lock for each of the parts.

#### Populations

The primary outcomes of both study parts will be analysed in the intention to treat (ITT) population. The ITT population consists of all randomised patients who have been exposed to the intervention (completed first infusion visit in NOR-DRUM B or completed second infusion visit in NOR-DRUM A). The per-protocol (PP) population will in each of the two study parts consist of all randomised patients who sufficiently comply with the protocol. Criteria for inclusion in the PP population will be specified in the SAP and the final criteria will be defined before database lock. The safety population consist of all patients who have been exposed to the intervention (same definition as the ITT population).

#### Statistical model

The primary outcomes will be analysed using logistic regression with treatment group as primary explanatory variable, adjusted for stratification factors used at randomisation. Although this is a multicentre study, study site will not be used for stratification or adjustment in the analysis due to anticipated small sample sizes within site. However, sensitivity analyses will be performed to assess the impact of site on the study conclusions. Other pre-specified covariates included in sensitivity analyses include age, use of disease-specific co-medication (methotrexate, azathioprine or similar) and levels of ADAb at baseline (NOR-DRUM B only). The SAP will detail these procedures, as well as alternative and further supportive evaluations, such as analyses including unbalanced baseline predictors or modifications of the logistic regression model in case validity assumptions are not met.

##### Primary analyses

The primary analysis will be performed on the ITT population. There will be two primary hypotheses tested in this study, one for each of the two parts (NOR-DRUM A and B). No adjustment for multiplicity will be made, since each part will be regarded as answering an independent research question.

In NOR-DRUM A, the statistical hypothesis tested is (superiority test): Null hypothesis: there is no difference in the proportion of patients in remission at week 30 between the intervention and control groups. Alternative hypothesis: there is a difference in the proportion of patients in remission at week 30 between the intervention and control groups. The hypothesis test will be evaluated by logistic regression analysis. A conclusion of superiority of either of the treatment strategies will be made if the null hypothesis is rejected at the 5% significance level. If the study fails to reject the primary null hypothesis, non-inferiority of TDM versus standard care will be assessed using a non-inferiority margin of 15%. Non-inferiority implies that the 95% confidence limits of the estimated adjusted risk difference of disease worsening lies fully within a non-inferiority margin of 15%.

In NOR-DRUM B, the statistical hypothesis tested is (superiority test): Null hypothesis: there is no difference in proportion of patients in sustained disease control throughout the study period (without disease worsening) between the intervention and control group. Alternative hypothesis: there is a difference in proportion of patients in sustained disease control throughout the study period (without disease worsening) between the intervention and control group. The primary hypothesis will be evaluated by the *p* value from the logistic regression analysis. A conclusion of superiority of either of the treatment strategies will be made if the null hypothesis is rejected on a significance level of 5%. If the study fails to reject the primary null hypothesis, non-inferiority of TDM versus standard care will be assessed, also using a 15% non-inferiority margin. Non-inferiority implies that the 95% confidence limits of the estimated adjusted risk difference of disease worsening lies fully within a non-inferiority margin of 15%.

##### Secondary analyses

Between-group comparisons will be performed for the primary endpoints on secondary populations in addition to secondary efficacy endpoints on both efficacy populations. The between-group comparisons for secondary variables will be tested as for the primary variable where applicable and additional analyses will be performed based on the following methods (but not limited to): repeated measures mixed models or appropriate non-parametric alternatives (continuous variables); logistic regression (possibly adjusting for within-subject dependencies by mixed model approaches) or Chi-square/Mantel–Haenszel test for binary response variables; Kaplan–Meier method (time-to-event variables) and comparisons between the two groups will be performed using the log rank test; and Cox regression analyses and/or appropriate parametric models such as the Weibull model. Methods to handle missing data may include complete case analyses, last observation carried forward, worst case/best case imputation and multiple imputation techniques. For the primary analyses, worst case imputation will be used for missing observations. Further details on missing data will be given in the SAP.

Safety analyses will be descriptive and presented as summary tables by treatment group and (if applicable) by visit. Patient-reported outcome measures (PROMs) and disability will be assessed using Short Form (36) Health Survey (SF-36) (generic), EuroQol 5 Dimensions (EQ-5D) (generic), MHAQ (RA, PsA, SpA), IBDQ (IBD) and DLQI (Ps). These scores will be summarised by descriptive summary tables at baseline and over time, and at the end of study. Missing data at end of study will be replaced by the last valid post-baseline assessment. We will perform subgroup analyses according to diagnoses groups (RA, SpA, PsA, UC, CD, Ps) on the appropriate primary and secondary variables using methods described above. Other exploratory subgroup analyses of primary, secondary and exploratory efficacy variables may be performed if appropriate. The decision to include such analyses will be made on basis of the collected data. Health economic analyses appropriate analyses as estimating the number of quality-adjusted life years (QALYs) obtained during the study period. For each patient, we will estimate one year’s costs based on registered data for utilisation of healthcare and the unit costs. The mean week QALYs and cost in the two treatment arms will be used to estimate an incremental cost-effectiveness ratio for all patients and according to diagnostic group.

### Adverse events

Any AEs encountered during the clinical study is reported in the eCRF. If the patient has experienced AE(s), the investigator records the following information in the eCRF: the nature of the event is described by the investigator in precise standard medical terminology. The duration of the event is described in terms of event onset date and event ended date. The intensity of the AE is graded as mild, moderate, severe, life-threatening or death. The causal relationship of the event to the study medication is assessed as unrelated, unlikely, possible, probable or definite. Events which are definitely due to disease progression are not reported as an AE. Serious adverse events (SAEs) are reported to the central study coordinating team.

### Data registration and monitoring

A web-based eCRF software solution is used to collect study data (Viedoc 4™, Uppsala, Sweden). The PI at each study centre is responsible for assuring that data entered into the eCRF is complete, accurate and that entry is performed in a timely manner. The electronic signature of the investigator will attest the accuracy of the data on each CRF. If any assessments are omitted, the reason for such omissions will be noted on the CRFs. Corrections, with the reason for the corrections, will also be recorded. A complete list of authorised study personnel will be maintained during the study and only study personnel authorised by the PI or coordinating investigator will be allowed to sign the eCRF. Protocol, protocol amendments, investigator’s brochure, informed consent and all study-related documents have been reviewed by an institutional review board and a GCP (Good Clinical Practice) certified person. All participating centres will be monitored during and after the trial by GCP-trained personnel in order to ensure compliance with GCP, the protocol and all other applicable regulations.

### Publications

Upon study completion and finalisation of the study report, the results of this study will be submitted for publication and posted in a publicly assessable database of clinical study results.

The results of this study will also be submitted to the Ethics Committee according to national regulations. All personnel who according to the ICMJE recommendations have contributed significantly in the planning and performance of the study will be included in the list of authors. Authorship will be based on scientific contribution and enrolment.

## Discussion

The clinical role of TDM of TNFi and other biopharmaceuticals used for immune mediated inflammatory disorders is still debated. Currently, implementation is based on clinical experience and observational studies, since only a few clinical trials have been conducted [24, 31]. To our knowledge, the NOR-DRUM study is the first RCT assessing the effectiveness and safety of TDM in INX treatment across a range of immune-mediated inflammatory disorders.

NOR-DRUM is investigator initiated and fully funded by a joint grant from the four Norwegian regional health authorities. The study is conducted as a shared effort by Norwegian rheumatologists, gastroenterologists and dermatologists, and 21 study centres across Norway participate in the data collection. Norway is particularly well suited to conduct this large scale trial on TDM. Due to a tender-based prescription system and to the early implementation of biosimilars, INX has, in recent years, been the preferred TNFi for Norwegian clinicians treating patients with immune-mediated inflammatory diseases. Additionally, the recent completion of the large NOR-SWITCH trial [32] has established a strong research collaboration between the different specialties using TNFi and good logistics for conducting clinical trials at the infusion units nationwide. Finally, the Department of Medical Biochemistry at Oslo University Hospital is a non-commercial, high-capability facility for antibody analyses and has developed assays for serum drug measurement of biological drugs and antidrug antibodies while performing these analyses on a self-cost basis.

The implementation of TDM depends on a validated therapeutic target range. The treatment algorithms in NOR-DRUM are based on an extensive literature review and expert opinions. They have been developed through a series of meetings in the project group consisting of national leading experts in this field (both clinicians experienced with TDM and laboratory physicians) and with additional input from international key experts in the scientific advisory board. The therapeutic level of INX is not definitely known for all the diseases, but there are strong indications that the lower limit is close to 3 μg/mL [20–25]. According to the literature review and expert opinion, the upper limit has been set to 8 μg/mL. The borders of the proposed therapeutic range, the yellow zones in Figs. 4 and 5, allow for some clinical considerations regarding the INX dosing. In the induction phase, the limits of 20 μg/mL at infusion 2 and 15 μg/mL at infusion 3 are based on clinical observations and previous literature [26, 33]. There is still no consensus whether dose adjustments or interval changes are the most effective and cost-effective way to increase or decrease the INX dose. Initial pharmacokinetic modelling suggested that a higher trough level could be achieved using less INX over time by shortening the interval instead of increasing the dose [34]. More recent studies suggest that a dose of, for example, 10 mg/kg every eight weeks is probably equal to 5 mg/kg every four weeks [35], and halving the infusion intervals are not superior to increasing dose when it comes to both effect and drug costs [36]. The proposed algorithms allows for both options, but due to lower drug costs in recent years, patient convenience and high costs of running infusion units, the preferred option is dose increase by increasing each infusion dose and dose decrease by increasing the infusion interval.

The NOR-DRUM study has an open-label design with no blinding of the patient or the study personnel. The disease activity measures included in the primary outcome are composite measures of patient-reported outcomes (PROs,) objective assessments and investigators assessments. Blinded outcome assessment was not considered feasible in this study, as it would have required both blinding of the patient and study personnel. Blinding of the study participants has not been possible as the participants in the intervention group experience changes in the dosing intervals based on serum drug levels. Feasible data collection within the frame of daily clinical practice at a large number of study centres has been important in this trial. Blinding of study personnel who do the outcome assessments would have required two sets of personnel at each site, making the study unfeasible. The open label design is a recognised weakness of the NOR-DRUM trial. Dose adjustments may occur in both arms though it is based on the study algorithm in the intervention arm only. Patients in both arms are treated with the same biological agent with standardised frequency of follow-up and the same management of complications. Sensitivity analyses will be performed to compare results of subjective and objective outcome measures.

The study is not powered to demonstrate superiority within each diagnostic group as inclusion of the required number of patients is not achievable in Norway within a reasonable timeframe and funding resources. As such, the primary endpoint is designed to evaluate the occurrence of remission (NOR-DRUM A) or disease worsening (NOR-DRUM B) across diseases. The choice of these generic endpoints are based on well-established measures of disease activity and predefined cut-offs for disease state and change for each diagnosis. The feasibility of these outcomes in a clinical-trial setting was tested successfully in the NOR-SWITCH trial [32]. The study is powered to detect a difference in remission rate of 15% (NOR-DRUM A) and a decrease in proportion of patients with disease worsening of 12.5% (NOR-DRUM B), which are both considered clinically meaningful differences.

In addition to assessing the effectiveness of TDM (the primary objective), we will also address safety, i.e. occurrence of infusion reactions and infections, and the effect of TDM on cost-effectiveness. Important exploratory objectives are predictions of immunogenicity. i.e. by genetic testing.

This large national multicentre trial assessing the effectiveness, safety and cost-effectiveness of TDM in patients treated with INX is expected to provide valuable information that will hopefully contribute to a reduction in the burden of disease in a range of common chronic diseases with a potentially disabling disease course, as well as a reduction of the high expenses related to biological therapy. The impact of TDM of biological therapy is currently a topic of great interest to clinicians, both nationally and internationally. Being the first trial to assess the effect of TDM in patients with a wide range of immune mediated inflammatory diseases on treatment with a TNF inhibitor, we hope the NOR-DRUM study will contribute to evidence-based implementation of TDM in standard care of patients treated with INX and other biological drugs.

## Trial status

Protocol version 1.2; December 2017.

Recruitment of patients started 1 January 2017 and will continue until the last patient has been included in NOR-DRUM B (expected 1 January 2020). Last patient’s visit in NOR-DRUM A was in October 2019. Last patient’s visit in NOR-DRUM B is estimated to be January 2021.

## Supplementary information

**Additional file 1.** SPIRIT 2013 Checklist: Recommended items to address in a clinical trial protocol and related documents.

## Data Availability

The full trial protocol (version 1.2 date 12 December 17) is available from the corresponding author on request. Data sharing is not applicable to this article as no datasets were generated or analysed during the current study.

## Abbreviations

ADAb: Anti-drug antibody(ies)
AE: Adverse event
ASDAS: Ankylosing Spondylitis Disease Activity Score
BASDAI: Bath Ankylosing Spondylitis Disease Activity Index
CD: Crohn’s disease
CDAI: Clinical Disease Activity Index
CRF: Case report form (electronic/paper)
DAS28: Disease Activity Score using 28 joints
DLQI: Dermatology Life Quality Index
DMARD: Disease-modifying anti-rheumatic drugs
eCRF: Electronic case report form
EQ-5D: EuroQol 5 dimensions
EULAR: European League Against Rheumatism
HBI: Harvey-Bradshaw Index
IBD: Inflammatory bowel diseases
IBDQ: Inflammatory Bowel Disease Questionnaire
IJD: Inflammatory joint diseases
INX: Infliximab
MHAQ: Modified Health Assessment Questionnaire
PASI: Psoriasis Area and Severity Index
PGA: Patient Global Assessment of Disease Activity
PhGA: Physician Global Assessment of Disease Activity
PMS: Partial Mayo Score
PRO: Patient-reported outcome
PsA: Psoriatic arthritis
PsAID: Psoriatic Arthritis Impact of Disease
QALY: Quality-adjusted life year
RA: Rheumatoid arthritis
RAID: Rheumatoid Arthritis Impact of Disease
SAE: Serious adverse event
SDAI: Simplified Disease Activity Index
SF-36: Short Form (36) Health Survey
SpA: Spondyloarthritis
SPIRIT: The Standard Protocol Items: Recommendation for Interventional Trials
TDM: Therapeutic drug monitoring
TNF: Tumor necrosis factor
TNFi: TNF inhibitor
UC: Ulcerative colitis
WPAI:GH: Work Productivity and Activity Impairment Questionnaire: General Health
